# Expert opinion in decision-making: a systematic review of methods and the INTEGRITY framework for incorporating expert consultation into research

**DOI:** 10.1186/s41073-026-00213-2

**Published:** 2026-06-24

**Authors:** Lesley Uttley, Louise Falzon, Nicholas Latimer, Thea Uttley, Shijie Ren

**Affiliations:** 1 School of Medicine and Population Health, University of Sheffield, Regent Court, 30 Regent Street, Sheffield, S1 4DA UK; 2 School of Mathematical and Physical Sciences, University of Sheffield, Hicks Building, Hounsfield Rd, Sheffield, S3 7RH UK

**Keywords:** Expert opinion, Systematic review, Research integrity, Expert consensus, Expert elicitation, Evidence-based decision-making, Psychosocial influences, Conflicts of interest, Bias, Methodological, Reporting

## Abstract

Experts and their opinions can play a pivotal role in research for decision-making, particularly where empirical data are limited or uncertain. However, the methods by which expert input is solicited, synthesised, and applied in research often lack consistency and transparency. The absence of cross-disciplinary guidelines for how expert opinion should be gathered, integrated into research, and utilised in decision-making leaves the process vulnerable to conflicts of interest, interpersonal dynamics, and researcher discretion. Using health technology assessment (HTA) as a case study, we conducted a systematic review of empirical studies examining the use of expert opinion in research for decision-making. Twenty-three studies were included, from which six broad categories of expert consultation methods were identified, ranging from informal individual consultation to structured expert elicitation protocols. Considerable variation was observed in both the methodological rigour and transparency with which expert consultation was conducted and reported. Drawing on the review findings, we propose a preliminary conceptual framework (INTEGRITY) that synthesises psychosocial, methodological and reporting factors to promote transparency, rigour, inclusivity and objectivity when incorporating expert consultation into research. For low-stakes exploratory judgements, informal consultation may be sufficient. For high-stakes policy or resource-allocation decisions that require quantitative estimates, structured and transparent approaches such as formal elicitation protocols should be considered to support inclusive, objective, rigorous, and transparent use of expert judgement in research.

**Supplementary Information:**

The online version contains supplementary material available at 10.1186/s41073-026-00213-2.

## Background

Decision makers frequently rely on expert input when decisions lack clear empirical answers (e.g., future risks, medical prognosis); or are politically or ethically sensitive (e.g., assisted dying); or require specialist interpretation of complex or disputed evidence (e.g., medical or forensic cases); or must be made under time pressure with limited data. The COVID-19 pandemic provided headline examples of panels of experts convened to form taskforces, making decisions on public health measures and interventions [1]. During this time, expert input in science was dissected and weaponised in numerous divisive debates that fuelled disinformation and misinformation campaigns regarding countless decisions and guidelines [2, 3]. Expert consultation is therefore no longer a peripheral methodological concern but is central to the legitimacy, transparency, and accountability of policy decisions, particularly in contexts where the stakes are considered high.

A particularly informative domain is health technology assessment (HTA), where expert judgements are used to estimate clinical and economic outcomes for new technologies in the face of conflicting stakeholder priorities [4]. HTA is a systematic, multidisciplinary process incorporating patient perspectives, as well as clinical experts to assess medical interventions, such as drugs, medical devices, and procedures to provide evidence-based information about the adoption, pricing, reimbursement, and utilisation of new health technologies. Decisions about which medical treatments to recommend and fund impact public health outcomes and resource allocation, therefore require transparency, accountability, and methodological rigour.

Despite the importance of expert input in areas of clinical uncertainty for HTA, the methodological standards guiding the practice of consulting experts remain inconsistent [5, 6]. Researchers in business and social psychology have long emphasised the importance of procedural fairness, accountability, and rational legitimacy in decision-making processes [7, 8]. Best practice guides are available for certain technical methods of expert consultation, such as multi-criteria group decision-making [9] or expert elicitation [10–12]. Yet, the majority of expert consultation in HTA often lacks detail on how experts are selected, how views are aggregated, and how methodological transparency is maintained to support replicability and stakeholder trust [13].

### Psychosocial factors: inclusivity and objectivity of experts

A number of psychosocial factors can influence how expert opinion is generated, interpreted, and incorporated into research and decision-making. This includes the credibility and selection of experts, interpersonal dynamics within expert groups, cultural and status hierarchies, cognitive biases, and conflicts of interest.

#### Inclusivity and selection of experts

**Fig. 1:**
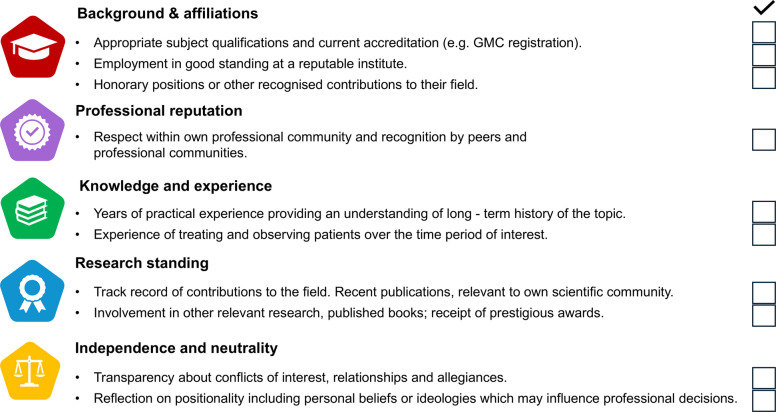
Key factors in determining professional credibility of experts for consultation in research

Experts are generally recognised through their knowledge, experience, and professional standing within a domain. However, consideration of what makes an expert credible and worthy of consultation is not universally standardised [14] and individuals who are highly visible or vocal within a field may not necessarily represent the diversity of informed opinion [15]. Ensuring that expert panels reflect a breadth of perspectives can strengthen the reliability and legitimacy of expert-informed research [16]. Multiple dimensions may contribute to perceptions of expertise, including academic achievements, professional experience, and recognition by peers. No single individual is likely to embody all relevant attributes and assembling panels that collectively encompass these qualities may help to mitigate potential gaps in expertise. Figure 1 summarises factors that researchers may consider when identifying credible experts for consultation in research.

#### Group dynamics when experts are convened

When convening experts for research to inform decision-making, various group dynamics can significantly influence the process and its outcomes. These dynamics can either enhance or subvert the quality of the collective judgement For example, groupthink may arise when pressure for consensus suppresses dissenting viewpoints [17] while social loafing may occur when individuals rely on others to contribute analytical effort. Information sharing biases can also lead groups to focus disproportionately on commonly held information while overlooking unique knowledge held by individual members [18]. Structured elicitation exercises and techniques such as Delphi processes can help to ensure that expert contributions are collected systematically and that dominant voices do not unduly shape group decisions [12, 19, 20].

#### Cultural and interpersonal differences

**Fig. 2:**
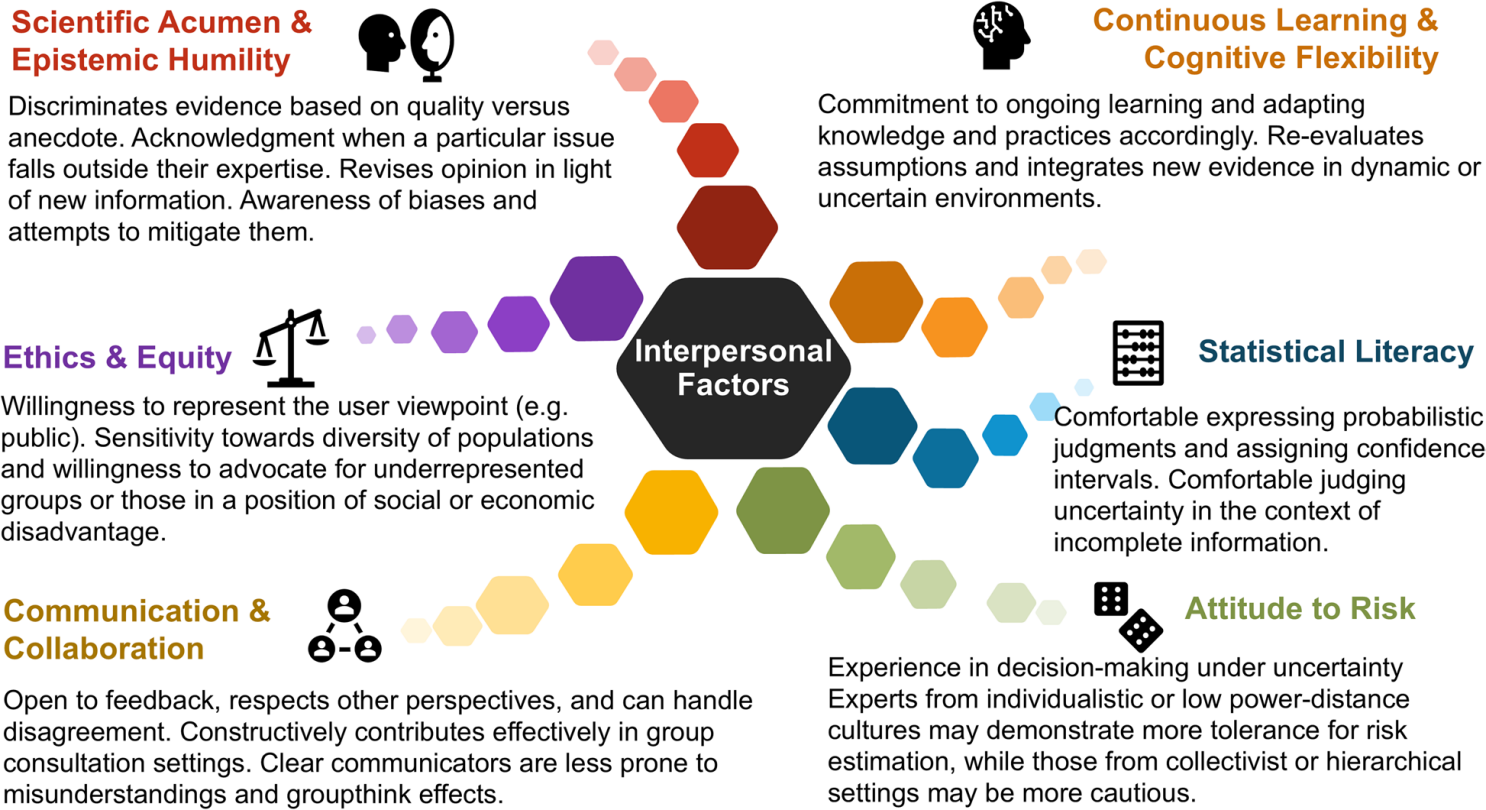
Interpersonal factors that may vary across experts recruited for input in research

Cultural factors may also influence how experts engage with consultation processes. Cultural norms shape how individuals interpret uncertainty, communicate expertise, and challenge the views of others. In individualistic cultures, experts may emphasise independent judgement and openly contest competing viewpoints [21]. In more collectivist or hierarchical contexts, maintaining group harmony or deference to senior figures may limit the expression of dissenting opinions. Similarly, hierarchical relationships within expert groups can influence participation, with junior experts sometimes reluctant to contradict more senior colleagues [22]. Personality differences may further shape contributions to expert consultations. More assertive individuals may dominate discussions, while quieter participants may contribute less visibly despite holding relevant expertise [23, 24]. Cultural and interpersonal diversity among experts can therefore enrich deliberation by bringing multiple cognitive perspectives to complex problems, provided consultation methods support balanced participation. Figure 2 summarises interpersonal and cultural factors that may influence expert interactions in research.

#### Heuristics and cognitive biases

**Fig. 3:**
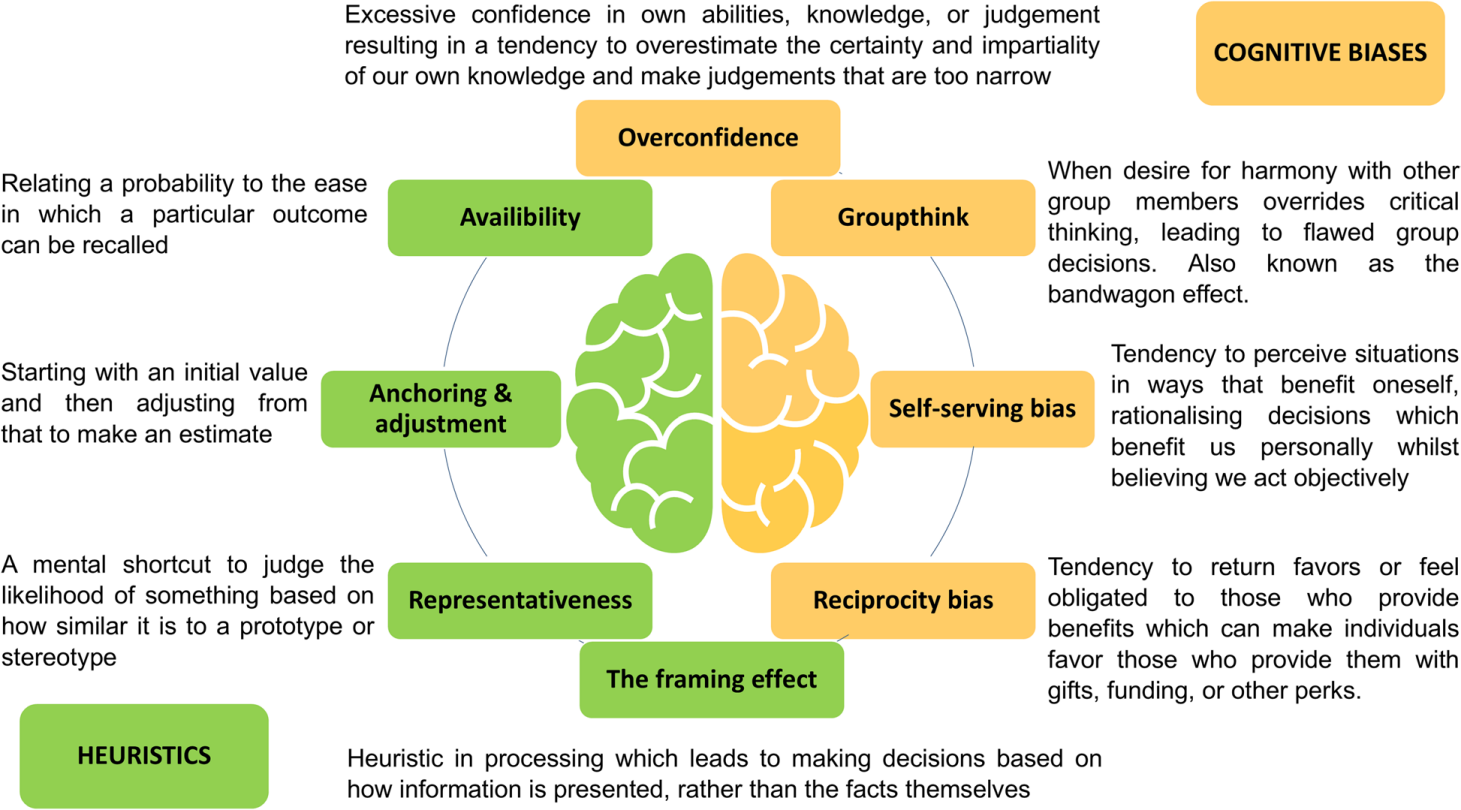
Heuristics and cognitive biases that can affect expert inputs in research

Even highly experienced experts remain susceptible to cognitive biases that affect judgement under uncertainty [20, 25]. Heuristics, or mental shortcuts used to simplify complex decisions, can lead to systematic deviations from rational reasoning. Examples include overconfidence, anchoring, availability bias, and confirmation bias. Emotional, motivational, and social influences may further shape how experts interpret information and estimate probabilities [26]. Figure 3 describes a range of commonly known heuristics and biases that can potentially be at play when consulting experts in research.

#### Conflicts of interest

Conflicts of interest represent another important consideration when incorporating expert opinion into research. Experts may have financial, professional, institutional, or intellectual interests that could influence their judgements. Many organisations therefore require declaration of conflicts of interest and may limit participation when conflicts are considered substantial. Recent European HTA regulations have also strengthened requirements for transparency and management of conflicts of interest among experts involved in joint clinical assessments [27].

**Fig. 4:**
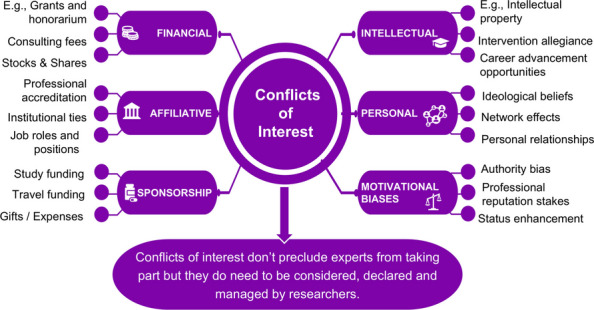
Potential conflicts of interest for experts in research

However, the presence of conflicts is not always straightforward to identify or manage. Interests may extend beyond direct financial ties to include professional allegiances, academic commitments, or reputational incentives. Researchers therefore play an important role in assessing and managing potential conflicts when assembling expert panels. Figure 4 illustrates common categories of conflicts of interest that may arise in research involving expert input.

Taken together, these psychosocial considerations highlight that expert consultation is shaped by both methodological choices and the social context in which experts operate. Decisions regarding expert selection, consultation format, facilitation, and reporting practices may all influence the quality and credibility of the resulting judgements. Transparent and systematic approaches to consulting experts are therefore essential to support the legitimacy of research used for decision-making, particularly in high-stakes policy contexts such as health technology assessment.

### Aims

Despite expert consultation being central to high-stakes decisions, particularly in health technology assessment where clinical, economic, and policy considerations intersect, clarity on methods for involving experts is lacking. A systematic review is needed to synthesise existing practices, identify gaps in transparency and rigour, and establish cross-disciplinary operational criteria that can improve the accountability, and trustworthiness of expert-informed research for decision-making.

The objectives of this research were:

1. To conduct a systematic review of empirical studies which examine the use of expert opinion in the process of health technology assessment and to conceptualise the different methods for eliciting expert opinion.
2. To synthesise the methodological characteristics, strengths, and limitations of these approaches and develop a conceptual categorisation of expert consultation methods used in HTA.
3. To consult methodological experts to obtain perspectives on the categorisation of methods identified in the review and to explore considerations relevant to their application in research.
4. To derive empirically informed methodological and reporting considerations that may support greater transparency, rigour, and research integrity when incorporating expert input into research.

## Methods

A protocol for this project was registered a priori with the Open Science Framework (https://osf.io/t94xk/).

### Systematic review of expert consultation in health technology assessment

#### Search strategy

Electronic bibliographic databases were searched by an experienced information specialist (LF) on August 8th, 2023, and updated on October 8th, 2024. Conceptual strings using all relevant subject headings and text words were combined using Boolean operators and translated into database specific syntax for: i. health technology assessment; ii. experts and; iii. opinion. The following biomedical electronic databases were searched: PubMed, Embase (Ovid), PsycINFO (Ovid), Epistemonikos and the Social Science Citation Index.

Literature searches were limited to articles in English, published after January 1995, when health technology assessment methodology was starting to be used and developed internationally. The full search strategies are provided in the supplementary appendices.

#### Supplementary searches

Additional records were identified by scanning the reference lists of relevant studies and reviews, by employing the Similar Articles feature in PubMed, and the Cited Reference Search in ISI Web of Science. The included studies of a technical report [12] and related review on a similar topic [6] were also examined for relevance.

#### Grey literature

Relevant websites were searched for non-published reports such as The International Network of Agencies for Health Technology Assessment (INAHTA), The National Institute for Health and Care Research (NIHR), The European Commission’s Public Health website, Overton and The King’s Fund.

#### Eligibility criteria

Inclusion criteria for this qualitative review question are defined using the SPIDER framework [28] (Sample, Phenomenon of Interest, Design, Evaluation, Research type) as detailed in Table 1.

**Table 1:** Eligibility criteria for studies relevant for the review

**Inclusion criteria**
**Sample**: Clinical or methodological experts **Phenomenon of Interest**: Use of expert opinion in the HTA process **Design**: Primary empirical studies **Evaluation**: Documenting the use of expert opinion in the process of health technology assessment **Research Type**: Articles published in the English language in peer-reviewed academic journals of studies published after the year 2000
**Exclusion criteria**
Discussion pieces or editorials without original primary data HTA agency documents/appraisals

#### Data management

Literature search results were imported from bibliographic databases into Covidence systematic review management software for de-duplication, screening and recording of reviewer decisions regarding inclusion/exclusion. Data from included studies were extracted into a bespoke Microsoft Excel data extraction template for each author.

#### Selection and data collection process

All citations were screened independently against eligibility criteria by two reviewers (LU and TU) in Covidence. Only studies which mentioned the use of expert input (or synonyms) in the title were included. Disagreements were resolved by discussion. Studies selected for full text inclusion were assessed for relevant data and quality assessment using Microsoft Excel. Data extraction of full text included papers was performed on all papers independently by two authors (LU and LF) and inconsistencies were resolved by discussion and consultation of the primary studies.

#### Data extraction

Relevant data items for extraction included: First Author name and year, URL to full text, Study type, purpose of research, clinical area, author team details, sample size (no. of experts), type of expert input (e.g., informal opinion, expert elicitation etc.), methods of expert input (e.g. survey, Delphi etc.), details on selection of experts where provided (e.g., qualifications, level of seniority etc.), contribution of opinion to research outcomes, characteristics of methods and reporting, strengths of method employed, weaknesses of method employed, funding information.

#### Outcomes and prioritisation

Eligible studies examine or document:

i. Primary outcome: Applied methods of integrating expert opinion in HTA
ii. Secondary outcome: Strengths and weaknesses of the applied methods of integrating expert opinion in HTA

#### Risk of bias/quality assessment: individual studies

Methodological studies do not yet have dedicated validated quality appraisal tools. In the absence of a formal dedicated tool to assess the reporting and methodological quality of included studies, the key criteria deemed relevant quality assessment criteria extracted in this review relate to 1) Conduct (availability of a study protocol indicating planning and pre-specification); 2) Reporting (whether the methods of the study are described in enough detail to allow replication).

#### Data synthesis

Extracted data were categorised by the type and method of incorporation of expert opinion. An overarching deductive framework was used to delineate the approaches of each method. Included studies are aggregated according to the type of expert consultation.

### Survey of methodologists on proposed ranking of methods expert consultation

#### Purpose of survey

A survey of research methodologists was conducted, as a form of consultation with the scientific community to sense-check the review’s conceptualisation of methods according to methodological rigour, and to gain further perspective on the respective strengths and weaknesses of methods in comparison to each other, that may not have been identified in the included studies. The survey was conducted through Qualtrics and ethical approval was granted from the University of Sheffield, School of Medicine and Population Health Ethics Committee.

#### Selection of survey sample

Potential participants were identified through their publications on meta-research integrity and identified as corresponding authors of studies included in a living methodological review on the problems with systematic reviews [29]. An invitation email was sent to the corresponding email addresses of authors of methodological studies, with an information sheet explaining how their details had been obtained for the study, politely requesting consideration to complete a short survey on the use of experts in research. Participants were informed that their responses would be anonymised and stored indefinitely as supplementary materials. Consent to participate was indicated by accessing the link to the survey.

#### Survey sample size

The number of methodological experts who were invited to participate in the survey via email was 80. An anticipated response rate of 15%–30% would yield an expected final sample of 12–24 respondents to inform the evidence-based research framework. As an exploratory and qualitative exercise, the aim was to receive a diverse range of opinion with the hope of achieving thematic saturation. Given the relevance of the survey topic to the research methods and the specialised nature of the target population, the survey intended to capture a manageable range of expert insights, providing tentative external validation of the categorisation of approaches research design.

## Results

### Systematic review results

**Fig. 5:**
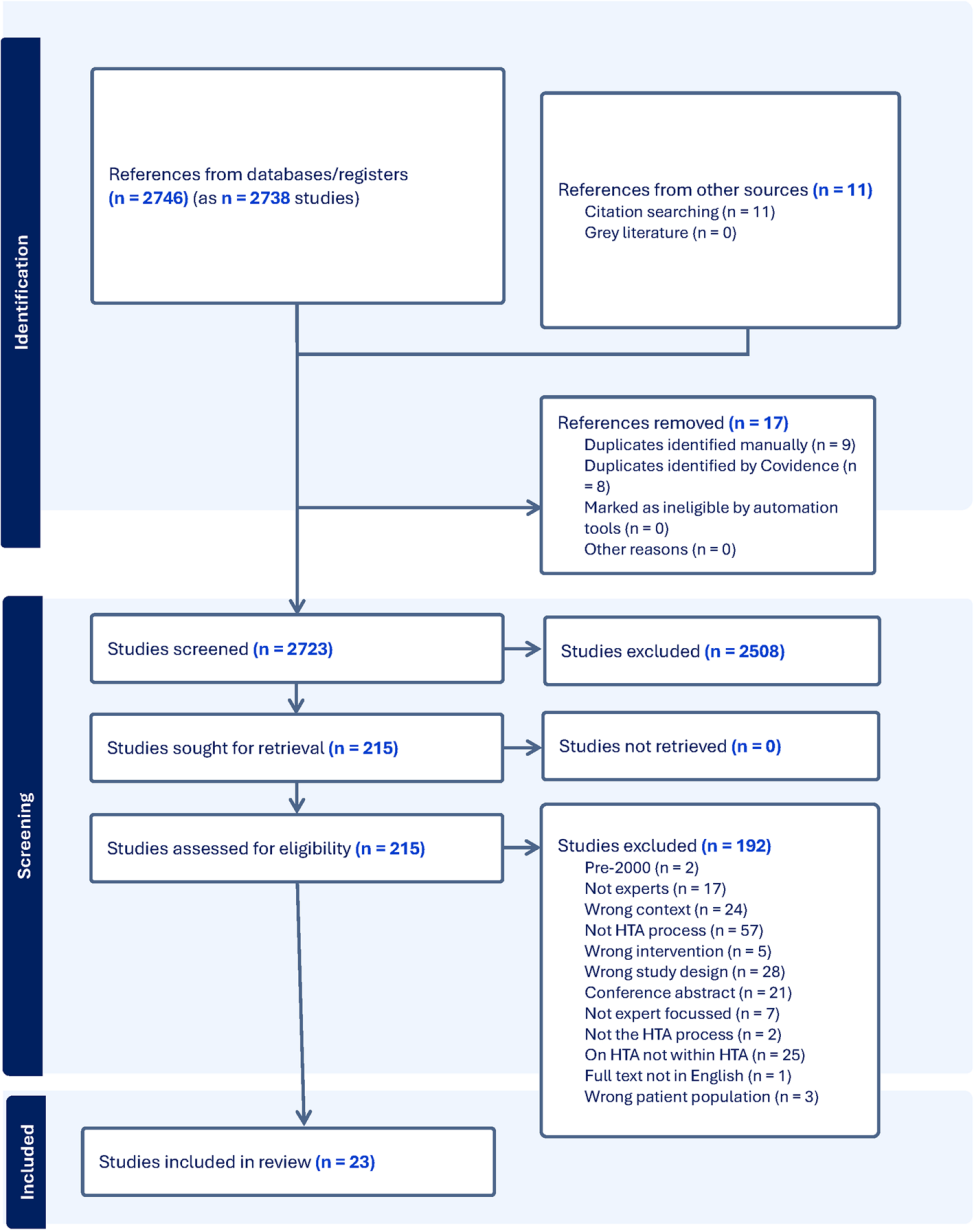
Flow diagram of studies retrieved, screened and included in the systematic review

The electronic bibliographic and supplementary literature searches generated 2746 citations and 2723 were screened at title and abstract stage once duplicates records were removed. 215 records were screened as full texts and 192 were subsequently excluded, as detailed in Fig. 5, leaving 23 articles that documented the use of expert input in HTA, that were included in the review.

#### Included studies

Details of the 23 studies included in the systematic review are described in Table 2.

**Table 2:** Details of 23 included studies in systematic review

**Study ID** **Journal**	**Study type** **Clinical area**	**Purpose of research**
Alemao et al., 2018 [30] PLOS One	HTA of current and novel interventions Rheumatoid arthritis	To inform a conceptual model for the cost-effectiveness of rheumatoid arthritis interventions
Angelis et al., 2020 [9] Social Science & Medicine	MCDA on treatment sequencing Prostate cancer	To gather experts' value preferences, including performance scoring and criteria weighting
Angelis et al., 2018 [31] Medical Decision-Making Policy & Practice	Expert preferences elicited for MCDA in HTA Metastatic prostate cancer treatments	To adapt and validate the value framework and assess the relative importance of different criteria in the model
Barone et al., 2014 [32] PLOS One	Consensus and Cost- Effectiveness Evaluation for Metastatic Colorectal Patients in an Italian Setting Oncology treatment	To consult on the implication of biomarker (KRAS) testing to optimize treatment of patients with metastatic colorectal cancer
Borsoi et al., 2023 [33] Global & Regional Health Technology Assessment	HTA of Radiofrequency Echographic Multi Spectrometry (REMS) in the diagnosis of osteoporosis Diagnostic musculoskeletal	To determine the costs of REMS vs. the conventional ionizing technology (dual-energy X-ray absorptiometry, DXA) for the diagnosis of osteoporosis
Grigore et al., 2016 [34] BMC Medical Research Methodology	Expert elicitation on HTA parameters Prostate cancer	To compare two elicitation methods on the same parameters and to collect subjective preferences of the experts
Grigore et al., 2017 [35] BMC Medical Informatics & Decision-Making	Methodological study of remote elicitation Primary care, Alcohol	To inform the development of a remote elicitation (EXPLICIT: EXPert eLICItation) tool by exploring the influence of mode of elicitation on elicited beliefs
Grimm et al., 2017 [36] Medical Decision-Making	Value of research analysis Preterm birth screening technology	To inform the parameters of a model of technology diffusion
Goodacre et al., 2018 [37] Health Technology Assessment	HTA of imaging pregnant or postpartum women with suspected pulmonary embolism Diagnostic imaging	To derive three new clinical decision rules for pregnant and postpartum women with a suspected pulmonary embolism
Gorelova et al., 2023 [38] Healthcare	Early Stage HTA Applied on Artificial Thymus for Patients with DiGeorge Syndrome Immunology	To compare two elicitation methods for populating conceptual decision-analytic models of transplantations in complete DiGeorge syndrome cases
Jankovic et al., 2022 [39] International Journal of Technology Assessment in Health Care	Structured expert elicitation on the extrapolated treatment effect from clinical trial data Geriatric physiotherapy	To extrapolate the treatment effect of a podiatry intervention to reduce the rate of falls and fractures in the elderly from clinical trial data
Knuttel et al. 2017 [40] Journal of Therapeutic Ultrasound	HTA Oncology treatment	To estimate cost-effectiveness of magnetic resonance-guided high intensity focused ultrasound ablation for the treatment of early-stage breast cancer
Lyratzopoulos et al., 2008 [41] International Journal of Technology Assessment in Health Care	Retrospective survey of expert clinician characteristics and their opinions NICE methods of HTA	To analyse a total of 598 expert clinician questionnaires relating to 182 different interventional procedures across the NICE HTA programme
Meads et al., 2013 [42] Health Technology Assessment	Elicitation of expert opinion to supplement diagnostic information needed for the economic evaluation Diagnostic imaging	To estimate cost effectiveness of positron emission tomography/computerised tomography imaging for recurrent cervical cancer
O'Callaghan et al., 2023 [43] Official Journal of the Irish Medical Organisation	Retrospective review of HTA submissions to National Centre for Pharmacoeconomics (NCPE) from July 2019 to June 2020 inclusive General medical submissions from Applicant Pharmaceutical Companies to NCPE (*n* = 18)	To review and document the use of clinical expert opinion to inform HTA submissions
Pandor et al., 2019 [44] Health Technology Assessment	HTA of treatment strategies for pharmacological thromboprophylaxis for lower-limb immobilisation after injury Injury/Haematology	To determine the most effective and cost-effective treatment strategy for pharmacological thromboprophylaxis for lower-limb immobilisation after injury
Petersohn et al., 2021 [45] Value in Health	Comprehensive uncertainty assessment in health economic modelling on the treatment of peripheral arterial disease Cardiology	Cost-effectiveness of treatment of patients with peripheral arterial disease (PAD) with dual platelet inhibition therapy (DPI) with rivaroxaban and aspirin
Pibouleau et al., 2014 [46] International Journal of Technology Assessment in Health Care	Methodological study to develop an Internet-based method for eliciting experts’ beliefs about the success rate of an intracranial stenting procedure Medical devices for intracranial aneurysms	To inform Bayesian priors for a model on intracranial neurostent success rates
Rossi et al., 2019 [47] Value in Health	Cost effectiveness analysis including expert elicitation for ultrasound Screening for renal cell carcinoma	To derive unknown quantities to inform a cost-effectiveness analysis
Singh et al., 2017 [48] International Journal of Technology Assessment in Health Care	Cost effectiveness methodological analysis Liver transplantation	To compare four approaches to estimate resource use in HTA when good quality contemporary data are not available
Soares et al., 2011 [49] Statistics in Medicine	Expert elicitation study for decision model Nursing Ulcer wound treatment	To elicit probabilistic beliefs on treatment effectiveness and healing rates
Soto-Mora et al., 2023 [50] International Journal of Technology Assessment in Health Care	HTA of caplacizumab Haemato-oncology	To determine the therapeutic classification of caplacizumab in treating acquired thrombotic thrombocytopenic purpura
Stevenson et al., 2020 [51] Health Technology Assessment	HTA of interventions to manage iatrogenic CJD Infectious disease control in surgery	To compare the cost-effectiveness of management strategies to minimise the risk of iatrogenic CJD from surgical instruments

#### Demographics of included studies

The majority of included studies were conducted in the United Kingdom setting [34, 35, 37, 39, 41, 42, 44, 47–51]. Two studies were conducted in The Netherlands [40, 45] two in Italy [32, 33]; one in Ireland [43]; one in the Czech Republic [38]; and one in Sweden. One was a multi-country study conducted in collaboration with HTA agencies and health insurance bodies in Sweden, Spain, Poland, and Belgium [31], one involved an international group of nineteen experts from Europe, North America, South America, and Asia [46]; one across thirteen countries in the European Union [9] and one was led by Bristol Myers Squibb consulting experts from a range of countries worldwide [30].

Studies were published between 2008 to 2023 and ranged in clinical focus between oncology, diagnostic imaging; infectious disease control; cardiology, rheumatology; primary care and injury management or multiple clinical areas across the HTA programme.

####  Quality of included studies

Study quality was broadly assessed by assessing rigour (whether methods were planned via a study protocol) and transparency (whether methods are reported in enough detail to permit replication), as detailed in Table 3. The full data extraction tables, including information on characteristics relating to the reporting completeness for all studies, are available to review at the Open Science Framework repository (https://osf.io/t94xk).

**Table 3:** Summary of quality assessment of included studies

Study ID	Planned	Methods described in enough detail to allow verification/replication
Alemao et al., 2018 [30]	No protocol	No
Angelis et al., 2020 [9]	No protocol	Many figures provided but not raw data
Angelis et al., 2018 [31]	No protocol	In principle but not in full
Barone et al., 2014 [32]	No protocol	In principle but not in full
Borsoi et al., 2023 [33]	Protocol was approved by the Ethics Committee of Università Commerciale Luigi Bocconi	In principle but not in full
Grigore et al., 2016 [34]	No protocol but ethics approval was obtained	In principle but not in full
Grigore et al., 2017 [35]	No protocol but ethics approval was obtained	No. Questions in questionnaire not provided
Grimm et al., 2017 [36]	No protocol	No
Goodacre et al., 2018 [37]	Protocol details plan for standard Delphi and Nominal Group methodology and ethics approval	Not all original data available unless through corresponding author
Gorelova et al., 2023 [38]	No protocol	Yes
Jankovic et al., 2022 [39]	Yes. Protocol appended as supplementary material	Yes, supplementary appendices provided
Knuttel et al., 2017 [40]	No protocol but ethics approval was provided	No. Questionnaire not provided
Lyratzopoulos et al., 2008 [41]	No protocol	In principle but not in full
Meads et al., 2013 [42]	Protocol for the broader HTA provided	Methods well reported but questionnaire not provided
O'Callaghan et al., 2023 [43]	No protocol	No
Pandor et al., 2019 [44]	Broader HTA protocol and PROSPERO registered	In principle but not in full
Petersohn et al., 2021 [45]	Yes. The elicitation protocol is available in the Appendix	In principle but not in full
Pibouleau et al., 2014 [46]	No protocol	In principle but not in full
Rossi et al., 2019 [47]	No protocol	Yes, full expert elicitation exercise, training material, and evidence dossier are found in the Appendix
Singh et al., 2017 [48]	No protocol but ethical clearance was provided	Supplementary materials to journal paper but behind paywall
Soares et al., 2011 [49]	No protocol	Not all original data available through supplementary material
Soto-Mora et al., 2023 [50]	No protocol	In principle but not in full
Stevenson et al., 2020 [51]	Broader HTA project protocol	Detailed documentation of the elicitation process

#### Use of expert input across studies

Across 23 included studies, six distinct methods of consulting experts in the HTA process were identified. These ranged from individual expert consultation through to structured expert elicitation, reflecting a gradient from informal, *ad hoc* input to highly formalised and replicable approaches. Methods for consulting experts in HTA were identified, as described in Table 4.

**Table 4:** Methods and description of expert consultation across studies

**Method** *Description*	**Included Studies Employing the Method**
**Individual expert consultation** *Consulting one or more experts without obtaining consensus or aggregating inputs*	O'Callaghan 2023 [43] (Mixed setting: interviews, questionnaires and advisory boards)
**Consultation of several experts individually e.g., survey, interview** *Consulting one or more experts with researcher defined aggregation*	Knuttel 2017 [40] (Remote: survey) Lyratzopoulos 2008 [41] (Remote: questionnaire) Alemao 2018 [30] (Setting not reported: Open-ended interviews)
**Expert consensus panel (in person)** *Experts aggregate their views openly with each other*	Goodacre 2018 (In person: Nominal group technique meeting) Pandor 2019 [44] (3rd Delphi in person round table) Angelis 2018 [31] (In person; group: Multi-criteria decision analysis (MCDA) MACBETH method) Angelis 2020 [9] (In person; group: MCDA MACBETH method)
**Delphi consensus (anonymous, remote)** *Researcher performs the aggregation of views; expert views remain anonymous*	Soto-Mora 2023 [50] (Remote, anonymous Delphi) Barone 2014 [32] (Remote, anonymous Delphi) Goodacre 2018 [37] (Remote; Individual: Delphi) Pandor 2019 [44] (Two rounds remote Delphi)
**Expert elicitation using an ad hoc or unspecified method** *Expert elicitation using fixed or variable interval techniques, to elicit and aggregate views of experts (e.g. histogram/chips and bins/roulette methods) but without specifying methods according to an established protocol*	Soares 2011 [49] (Individual [assume in person]: histogram method) Meads 2013 [42] (In person; group training/individual elicitation: allocation of points) Singh 2017 [48] (Remote; individual: Excel tool based on Leal 2007) Grigore 2016 [34] (Face-to-face; individual: Histogram vs hybrid methods) Rossi 2019 [47] (Face to face or telephone; individual: quantile method) Borsoi 2023 [33] (Remote; individual: Excel tool: point estimates) Gorelova 2023 [38] (In person; individual: comparison of fixed interval & histogram methods) Grigore 2017 [35] (In person vs remote; individual: EXPLICIT tool) Pibouleau 2014 [46] (In person vs remote; individual: histogram method) Goodacre 2018 [37] (Setting not reported: ad hoc expert elicitation) Grimm 2017 [36] (Setting not reported: linear pooling)
**Structured expert elicitation** *Uses structured techniques such as fixed-interval or variable interval techniques (e.g. histogram/chips and bins/roulette methods) and reports methods using an established protocol (i.e. Cooke’s classical method; IDEA protocol; MRC reference protocol; Sheffield Elicitation Framework (SHELF)*	Stevenson 2020 [51] (In person; group SHELF method) Jankovic 2022 [39] (Remote and in person; individual: roulette method) Petersohn 2012 [45] (Remote; individual: EXPLICIT tool)

#### Categories of methods of expert consultation across included studies

**Fig. 6:**
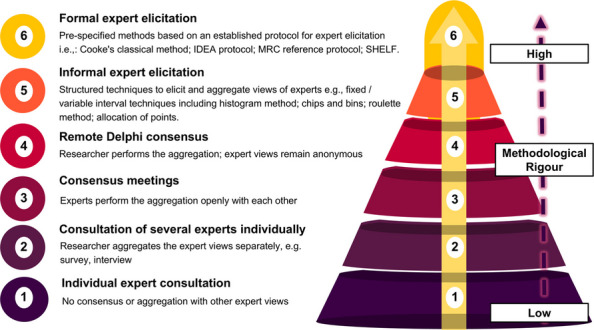
Proposed hierarchy of methods of incorporating experts into research according to methodological rigour

The use of the six categories of methods across studies regarding their reported strengths and drawbacks are described below and depicted in a tentative hierarchy according to methodological rigour in Fig. 6.

i. Individual expert consultation was the simplest approach, involving interviews, questionnaires, or advisory boards with single experts or small groups. While flexible and quick to implement, this method often lacked transparency in how experts were selected and how their judgements were integrated into final decisions.
ii. Consulting multiple experts with researcher-defined aggregation added analysis via synthesis of inputs, but aggregation procedures (e.g., averaging responses) were often opaque, limiting replicability and leaving space for researcher bias and selective reporting.
iii. Expert consensus panels convened in person. These methods enabled deliberation and joint interpretation, but carried well-documented risks of dominant voices, groupthink, and hierarchical deference. This category includes Multi-Criteria Decision Analysis (MCDA) which applies structured frameworks for aggregating expert preferences, especially in contexts requiring explicit trade-offs across competing criteria. MCDA can promote transparency but are resource-intensive and not always feasible in applied HTA contexts.
iv. Delphi methods, particularly remote and anonymous variants, offer a way to mitigate interpersonal pressures while allowing iterative refinement of judgments. However, the quality of Delphi applications varied, with some studies failing to report key details about iteration procedures, attrition, and stopping rules.
v. Expert elicitation using ad hoc methods (e.g., histogram or roulette techniques) reflected attempts to quantify uncertainty but frequently omitted clear protocols or justification for chosen techniques, reducing reproducibility.
vi. Structured expert elicitation protocols (e.g., Cooke’s Classical Method, IDEA, SHELF) represented the most rigorous tier, emphasising training, calibration, and explicit aggregation rules. Only a minority of studies employed such approaches, but these offered the strongest safeguards against bias and provided detailed reporting for replicability.

### Results of survey with methodologists

From an invitation sent to 80 corresponding authors on two occasions in June and July of 2025, 18 respondents began the survey but only 11 respondents completed the survey. Full details of the survey, responses and analyses are provided in the supplementary appendix. In brief, the survey asked whether respondents had experience of either conducting or participating in any of the six methods identified; whether they agreed with the proposed order of methods according to methodological rigour as outlined in Fig. 6 above (with “1” indicating the lowest rigour and “6” indicating the highest rigour) and whether they had any other thoughts on the strengths and drawbacks on the six methods.

When looking at the mode of the rankings in Table 5 alone, five of the six proposed categories received the same modal rank as suggested in the hierarchy proposed by the project team. However, agreement among respondents overall was very low (Kendall’s W = 0.04) and well below statistical significance, χ^2^ (5) = 2.27, *p* = 0.81. Given the lack of consistent agreement, and the low number of respondents (*n *= 11), we do not consider the survey results to provide confirmatory validation or endorsement for the proposed ranking of methods. The feedback on strengths and drawbacks from the survey (provided in supplementary appendix) provided further context that study authors are likely mindful of time constraints, particularly where answers are needed quickly, and that these are likely to supersede aspirations to perform the most methodologically rigorous approach available in many decision-making settings. Moreover, methods which may be regarded as more rigorous and formulaic such as expert elicitation were more likely to be downgraded by survey respondents when the specific methods were not reported well as this lack of transparency from study authors was seen as less likely to indicate trusted research. Transparency was therefore seen as a necessary component to explicitly demonstrate rigour in this regard.

**Table 5:** Ranking of methods according to methodological rigour by 11 survey respondents

Method	Proposed Ranking	Mode (Survey)
Individual expert consultation	1 (Lowest)	1
Consultation of several experts individually	2	2
Consensus meetings (face-to-face)	3	5
Delphi consensus (remote, anonymous)	4	4 & 6
Expert elicitation (full methods not specified)	5	5
Expert elicitation (full methods specified)	6 (Highest)	6

### Methodological and reporting characteristics for expert consultation in research

The process of extracting data across the included studies revealed key factors that differed across studies which may moderate the outputs when consulting experts. These have been collated and organised chronologically to provide a taxonomy of methodological and reporting items when consulting experts (Tables 6 and 7). Details regarding the selection, recruitment and demographic details of experts are particularly variable across the included studies. Factors related to the professional credibility, interpersonal characteristics or cultural factors were seldom reported across all studies (see OSF repository (https://osf.io/t94xk) for data extraction tables). Additionally details regarding how potential conflicts of interest, biases and heuristics arising from the process were frequently absent. Process details regarding whether experts were provided with training, calibration, information or evidence dossiers during consultation were scant as well as whether the intention was to consult experts inductively (before providing information) or deductively (after sharing information). The methodological factors identified (Table 6) may be used by future researchers assessing the methodological conduct used in studies documenting expert consultation as indicators of rigour.

**Table 6:** Methodological factors for researcher and organisations to consider in the process of consulting experts

	**Label**	**Justification**
M1	Expert selection	Describe the criteria used to identify and select experts, including indicators of professional credibility such as qualifications, experience, or recognised expertise
M2	Demographics	Describe relevant characteristics of the expert panel, including disciplinary background, geographic representation, or other attributes that may influence the diversity of perspectives
M3	Best Practice	Specify whether an established method, protocol, or framework for expert consultation was used, and provide the relevant citation where applicable
M4	Bias Appraisal	Describe any measures used to identify or mitigate potential cognitive, motivational, or group biases that may influence expert judgements
M5	Pre-specification	Report whether the expert consultation methods were prespecified in a protocol and provide access to the protocol where available
M6	Ethics approval	Report whether ethical approval or oversight was obtained for the expert consultation and identify the approving body where relevant
M7	Prior knowledge of data	Describe the timing of expert consultation relative to exposure to research evidence or materials (e.g., before or after reviewing background information or evidence summaries)
M8	Facilitator	Describe the role of any facilitator or moderator involved in the expert consultation and how they supported balanced participation
M9	Setting	Describe the mode and setting of the consultation, including whether interactions occurred in person, remotely, or through mixed formats
M10	Calibration and training	Describe any training, calibration exercises, or preparatory activities provided to experts prior to consultation
M11	Researcher Synthesis	Describe how expert inputs were synthesised or aggregated, including any mathematical, statistical, or qualitative approaches used by researchers
M12	Consistency	Describe how expert contributions were treated within the consultation process, including whether inputs were weighted equally or differentially
M13	Reflexive Aggregation	Describe whether experts had the opportunity to review or revise their responses after exposure to the views of other participants
M14	Timeline	Describe the timing and structure of the consultation process, including the number of rounds or phases where applicable
M15	Face validity	Describe any procedures used to assess whether the consultation outputs were considered by experts to accurately reflect their views
M16	Validation	Describe any approaches used to validate expert inputs against other data sources, evidence, or analytical procedures
M17	Resource Use	Describe the resources required to conduct the expert consultation, including time commitments, tools, or software used
M18	Reimbursement	Report any compensation, reimbursement, or acknowledgment provided to experts for their participation
M19	Transparency	Describe how methodological transparency was ensured, including availability of consultation materials, instruments, or protocols
M20	Funding	Report the sources of funding and any financial support associated with the expert consultation process

**Table 7:** Reporting characteristics researchers should consider for consulting experts

	Label	Description
R1	Expert selection	The process used to identify, select and recruit experts should be described.
R2	Demographics	Relevant characteristics of participating experts, such as demographics, equity characteristics, disciplinary background, or geographic context should be reported where appropriate.
R3	Best Practice	Any model, methodological framework, or checklist guiding the expert consultation should be cited or referenced.
R4	Pre-specification	Information on protocol registration or access to a publicly accessible study protocol should be provided where applicable.
R5	Ethics approval	Ethical approval and oversight arrangements, including the approving body where relevant, should be reported.
R6	Facilitator	The presence and role of any facilitator or moderator involved in the consultation process should be described.
R7	Calibration and training	Any training, calibration exercises or orientation provided to experts to support consistent understanding should be described and justified.
R8	Consistency	The approach used to treat expert contributions, including whether inputs were weighted or treated equally, should be described and justified.
R9	Prior knowledge of data	Any background information, evidence dossier or literature provided to experts prior to consultation should be described.
R10	Reflexive Aggregation	Where experts are able to influence or revise group estimates, the process by which expert views interact or are iteratively updated should be clearly reported.
R11	Researcher Synthesis	The method used to synthesise experts, such as averaging, weighting, or applying decision thresholds, should be clearly reported.
R12	Setting	The mode and setting of consultation, including whether interactions occurred in person, remotely, or through mixed formats should be stated.
R13	Timeline	The timing and sequence of expert consultation activities, including the number of rounds or phases where applicable, should be reported.
R14	Face validity	Any procedures used to assess or confirm that consultation outputs accurately reflect expert views should be described.
R15	Bias Appraisal	Measures taken to identify or mitigate potential cognitive biases, conflicts of interest, or other influences on expert judgement should be reported.
R16	Validation	Any approaches used to validate expert inputs against other data sources, evidence, or analytical procedures should be described.
R17	Resource Use	The resources, tools, or software required to conduct the consultation process should be reported where relevant.
R18	Reimbursement	Any compensation, reimbursement, or authorship arrangements relating to expert participation should be disclosed.
R19	Transparency	Information on data sharing, availability of elicitation materials, or access to consultation outputs should be provided where possible.
R20	Funding	Sources of funding and any relevant financial support for the study should be reported.

Characteristics that represent reporting completeness in studies of experts used in research are reported in Table 7.

## Discussion

This study combines a systematic review of methods for incorporating expert consultation into research, with the development of a conceptual framework to support their use in practice. The review identified 23 empirical studies of expert consultation in HTA which identified key characteristics relating to methodological rigour and reporting transparency. Informal and ad hoc methods dominate current practice, suggesting that ease and flexibility are often prioritised over reproducibility and transparency but such methods risk embedding biases, interpersonal influences, and unacknowledged uncertainties into decision-making. At the other end of the spectrum, structured expert elicitation protocols provide the opportunity to conduct high-quality methodological research which converts expert input formally into data, but such methods are less commonly used in HTA, possibly due to their speciality, training requirements, and resource intensity. Moreover, when they are used, they are often reported with insufficient detail to assess their methods or to enable other researchers to replicate them. Methods which maintain some anonymity and consistency such as Delphi, occupy an intermediate space, balancing feasibility and transparency for reaching consensus on qualitative topics, however they require detailed, transparent reporting to avoid undermining their credibility.

**Fig. 7:**
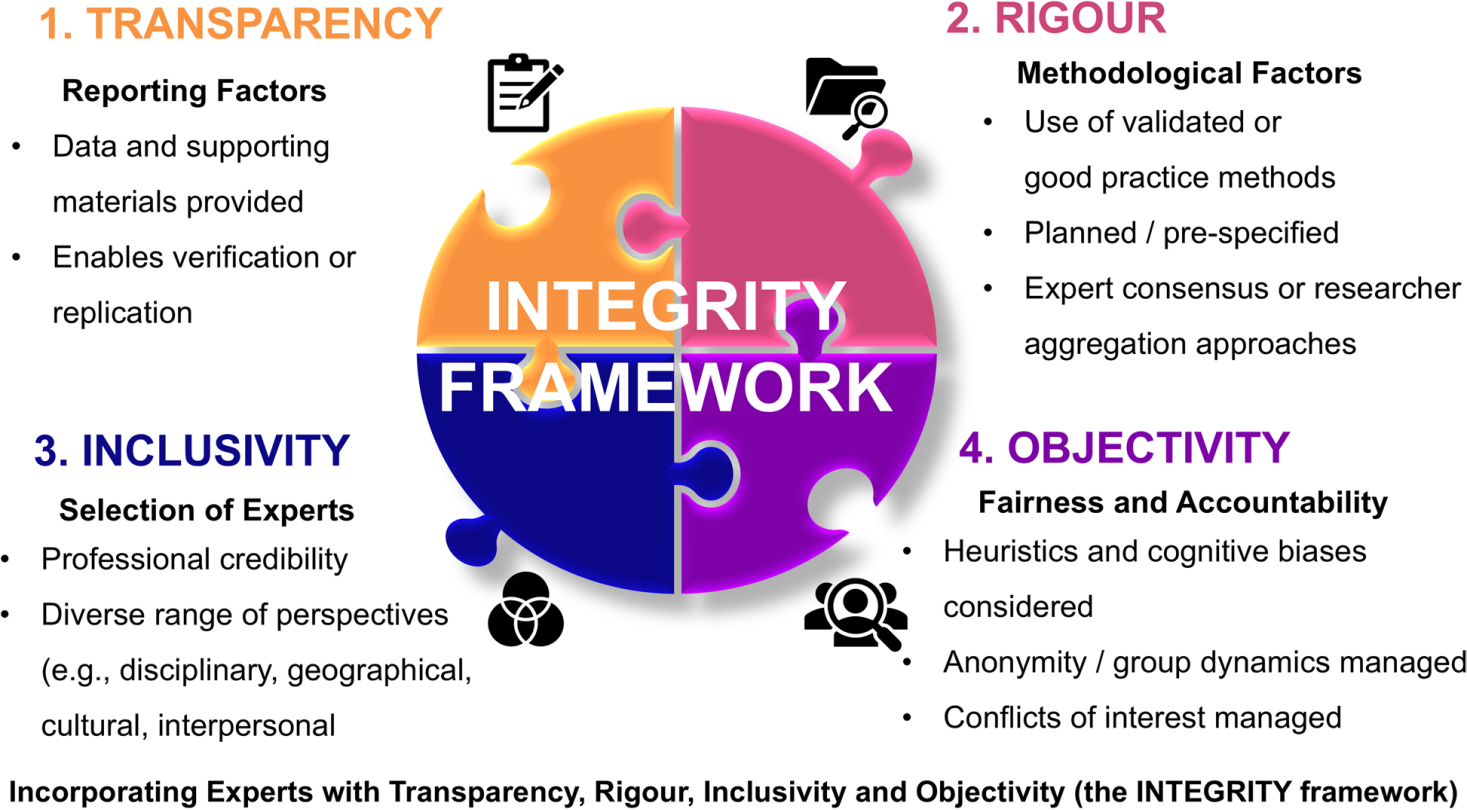
Incorporating Experts with Transparency, Rigour, Inclusivity and Objectivity (the INTEGRITY framework)

The evidence retrieved in this systematic review highlights the lack of consistency in how expert input is gathered, reported, and integrated into research and decision-making. By delineating a structured set of 20 methodological and 20 reporting characteristics, we provide preliminary guidance to support researchers, organisations, and decision-makers in incorporating expert opinion into research with greater consideration of inclusivity, transparency, rigour, and objectivity in research involving experts. These characteristics underpin how experts are selected and how the process of involving them can be managed according to principles outlined in the introduction with regards to professional credibility, interpersonal or cultural factors, heuristics or biases and conflicts of interest. To synthesise these considerations, we propose the INTEGRITY framework, which organises the key factors identified in this review into four domains relevant to research integrity when incorporating expert consultation into research (Fig. 7).

We have proposed a conceptual framework termed INTEGRITY. Transparency in reporting expert consultation ensures credibility and reproducibility; Rigour in planning and prespecifying upholds methodological conduct and best practice; Inclusivity in expert selection ensures an appropriate range of credible experts are consulted; Objectivity ensures the act of involving selected experts preserves research independence and fairness. The elements presented in this review help to safeguard against bias, promote diverse representation of contributors, and enable critical appraisal of research that incorporates experts. These general points for guidance may serve as a transferable model for decision-making contexts where expert judgement is essential.

**Fig. 8:**
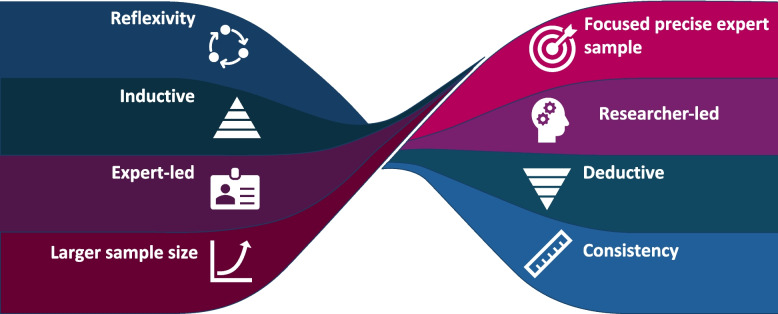
Tensions and trade-offs in methods choices for consulting experts

The survey did not demonstrate clear agreement among respondents regarding the ranking of methods according to methodological rigour, highlighting the subjective nature of such assessments and reinforcing the importance of transparent reporting rather than reliance on any single hierarchy. The categories identified in the proposed hierarchy may provide clarity to researchers and decision makers on the range of consultation methods available which may support choice proportionate to the type of input solicited, such as formal quantitative estimations, as opposed to general qualitative consensus, according to the risk of the decision problem. The hierarchy of methods was proposed with a focus on research integrity however upholding rigour is not necessarily the aim for everyone conducting expert consultation in research for decision-making. Other aims could include to gain perspectives, to seek validation or to increase clout of conclusions. The needs of the decision problem, time constraints, resources and experience within the research team likely govern which method of consultation is preferred. There are various methodological trade-offs that researchers may choose between that can serve as potential moderators of the output received (for examples, see Fig. 8).

As highlighted in feedback from our survey with methodological experts, time constraints, particularly where answers are needed quickly likely govern how experts are often consulted. For low-stakes, exploratory judgements, more informal methods may suffice. However, for high-stakes policy or resource-allocation decisions that require numerical estimations, rigorous and transparent methods such as structured elicitation protocols should be considered.

### Limitations

This review was limited to literature published in peer-reviewed academic journals. Inclusion of unpublished reports from HTA agencies may well have provided further in-depth information on what happens in practice. To explore further nuances, future research could survey HTA agencies and conduct literature review of grey literature to provide further insights as to how and why differential approaches are used when incorporating experts into research and their relative strengths and weaknesses. In the survey of methodological experts, whilst 18 respondents began the survey, only 11 respondents completed it, limiting the final sample of responses, and therefore the validation on the proposed categorisation of methods. The methodological and reporting criteria were proposed based on the empirical literature retrieved and refined by authors to confirm that each were conceptually relevant to at least one method of expert consultation. As a preliminary framework they are suggestions to improve planning, conduct and reporting, rather than prescriptive. Although informed by HTA literature, further work across other decision-making domains is needed to assess generalisability. Future research may apply and further refine the INTEGRITY framework across diverse decision-making contexts to evaluate its usefulness for strengthening transparency and research integrity when incorporating expert judgement.

## Conclusion

This research identifies key psychosocial, methodological, and reporting considerations that can shape how expert input is incorporated into research. From these findings we outline the INTEGRITY framework, which highlights the importance of inclusivity, objectivity, methodological rigour, and transparency when consulting experts in research. These considerations may assist researchers and organisations in incorporating expert judgement into research in ways that strengthen the integrity and credibility of expert consultation used for decision-making under uncertainty.

## Supplementary Information

Supplementary Material 1.

## Data Availability

The datasets supporting the conclusions of this article are available as supplementary appendices and at (https://osf.io/t94xk/).
