# Supplementary material for: Expert opinion in decision-making: a systematic review of methods and the INTEGRITY framework for incorporating expert consultation into research
Source: Res Integr Peer Rev. 2026 Jun 24;11:25. doi: 10.1186/s41073-026-00213-2 (PMC13292522; doi:10.1186/s41073-026-00213-2)
Supplement: Supplementary file 1 — Supplementary Material 1. [file 41073_2026_213_MOESM1_ESM.docx]

**Expert opinion In Health Technology Assessment Appendix**

PROTOCOL, FULL DATA EXTRACTION TABLES AND SURVEY RESPONSES AVAILABLE AT : <https://osf.io/t94xk/>

# Search Strategies

All databases were searched on August 8^th^, 2023 and updated on October 8^th^, 2024

**PubMed**

#1 Technology Assessment, Biomedical[MeSH]

#2 technology assess*[Title/Abstract] OR hta[Title/Abstract]

#3 #1 OR #2

#4 Health Personnel[MeSH]

#5 clinician*[Title/Abstract] OR physician*[Title/Abstract] OR expert*[Title/Abstract] OR advisor*[Title/Abstract] OR consultant*[Title/Abstract]

#6 #4 OR #5

#7 Opinion*[Title/Abstract] OR Elicit*[Title/Abstract] OR consensus[Title/Abstract] OR input[Title/Abstract]

#8 #3 AND #6 AND #7 **English, from 1995 - 2024**

**Embase (Ovid)**

Original search: 1995 to 2023 Week 31 Update search: 2023 to 2024 Week 40

**1**  exp biomedical technology assessment/
**2**  (technology assess* or hta).tw.
**3**  1 or 2
**4**  exp health care personnel/
**5**  (clinician* or physician* or expert* or advisor* or consultant*).tw.
**6**  4 or 5
**7**  (Opinion* or Elicit* or consensus or input).tw.
**8**  and/3,6-7
**9**  limit 8 to (english language and yr="1995 -Current")

**PsycINFO (Ovid)**

Original search: 1995 to July Week 4 2023 and Update search: 2023 to 2024 Week 5

**1**  (technology assess* or hta).tw.
**2**  exp health personnel/
**3**  (clinician* or physician* or expert* or advisor* or consultant*).tw.
**4**  2 or 3
**5**  (Opinion* or Elicit* or consensus or input).tw.
**6**  and/1,4-5
**7**  limit 6 to (english language and yr="1995 -Current")

**International HTA Database (INAHTA)**

#1 "Biomedical Research"[mhe]

#2 technology assess* OR hta[all fields]

#3 #1 OR #2

#4 "Health Personnel"[mhe]

#5 clinician* OR physician* OR expert* OR advisor* OR consultant*[all fields]

#6 #4 OR #5

#7 Opinion* OR Elicit* OR consensus OR input[all fields]

#8 #3 AND #6 AND #7 Limited to 1995 to 2024, English

**Epistemonikos**

(title:((title:(technology assess* OR hta) OR abstract:(technology assess* OR hta)) AND (title:(clinician* OR physician* OR expert* OR advisor* OR consultant*[) OR abstract:(clinician* OR physician* OR expert* OR advisor* OR consultant*[)) AND (title:(Opinion* OR Elicit* OR consensus OR input) OR abstract:(Opinion* OR Elicit* OR consensus OR input))) OR abstract:((title:(technology assess* OR hta) OR abstract:(technology assess* OR hta)) AND (title:(clinician* OR physician* OR expert* OR advisor* OR consultant*[) OR abstract:(clinician* OR physician* OR expert* OR advisor* OR consultant*[)) AND (title:(Opinion* OR Elicit* OR consensus OR input) OR abstract:(Opinion* OR Elicit* OR consensus OR input)))) Dates 1995 - 2024

**Social Science Citation Index**

#1 **TS=(technology assess* OR hta)**

**#2** TS=(clinician* OR physician* OR expert* OR advisor* OR consultant*)

#3 TS=(Opinion* OR Elicit* OR consensus OR input)

#4 #1 AND #2 AND #3 from 1995, English language

**Grey Literature Searching**

The following sites were searched on August 16^th^, 2023

The International Network of Agencies for Health Technology Assessment (INAHTA) (https://www.inahta.org/)

The National Institute for Health and Care Research (NIHR) (https://www.nihr.ac.uk/research-funding/funding-programmes/health-technology-assessment)

The [European Commission’s Public Health website](https://health.ec.europa.eu/health-technology-assessment_en) (https://health.ec.europa.eu/health-technology-assessment_en)

Overton (<https://www.overton.io/> via University of Sheffield)

The King’s Fund (https://www.kingsfund.org.uk/)

**Included Studies from the following report were checked for relevance:**

Oakley J. E., Ren S., Forsyth J. E., Gosling J. P., Wilson K., Latimer N., Rutherford M. J., Uttley L., Fotheringham J., NICE DSU Technical Support Document 26: Expert elicitation for long-term survival outcomes. 2025. Available from <http://www.nicedsu.org.uk>

# Survey Content

Use of expert opinion is common in healthcare decision making, particularly where there is uncertainty in the evidence base. We're conducting a review on expert opinion in decision making and we'd like to explore your views on the methodological benefits and drawbacks of using expert opinion in such work.

This is a very short survey on your thoughts on the use of expert opinion and a proposed framework of expert opinion in research, which we'd be very grateful if you have time to complete. Your input will be used to propose a general methodological framework for incorporating expert opinion into research.

From reviewing the academic literature of incorporating expert opinion using health technology assessments as a case study, the use of expert opinion in evidence-based decision making broadly falls into six categories outlined in the survey.

Have you had experience of the below methods in the context of evidence-based decision making (either in person or remotely)?


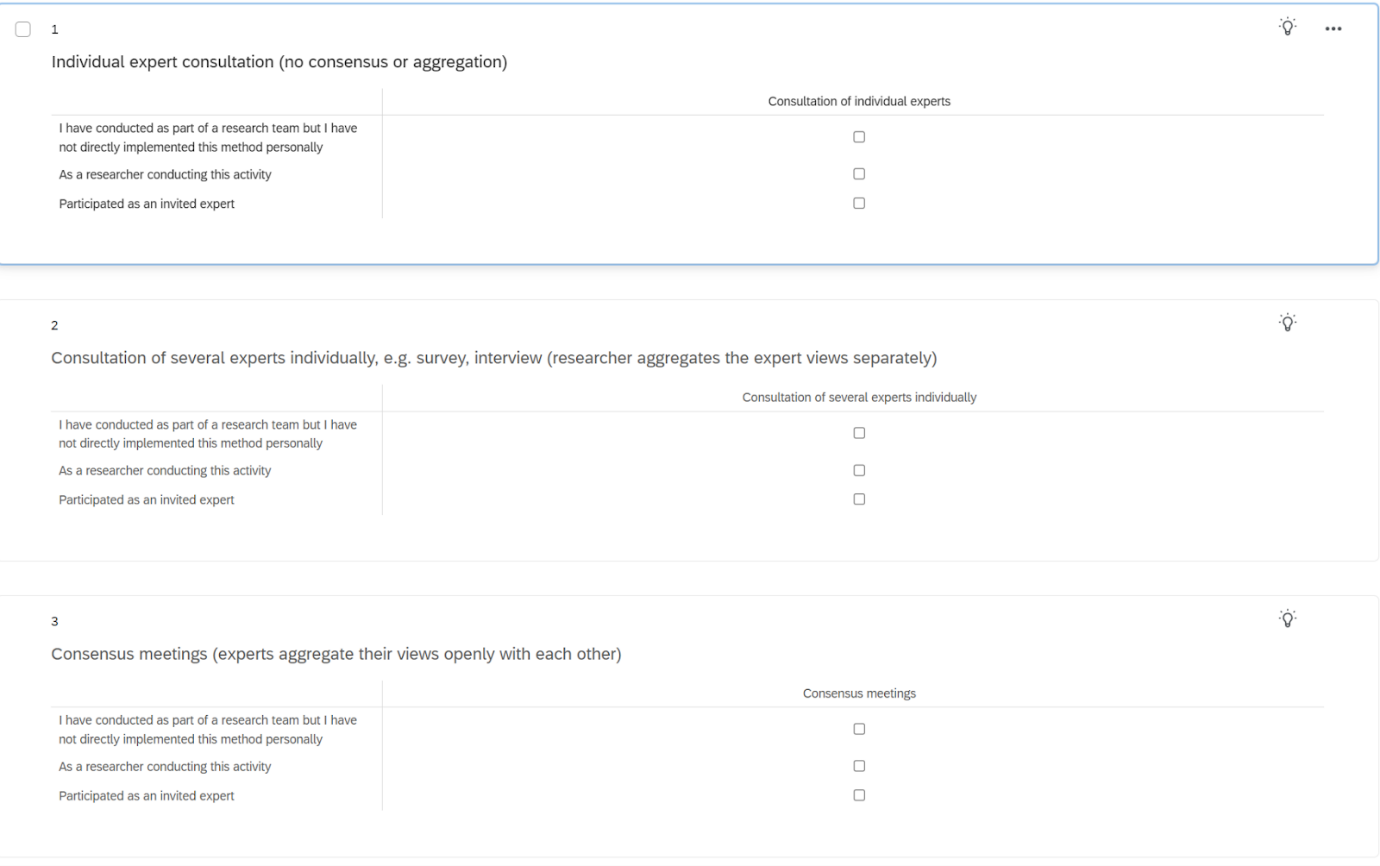


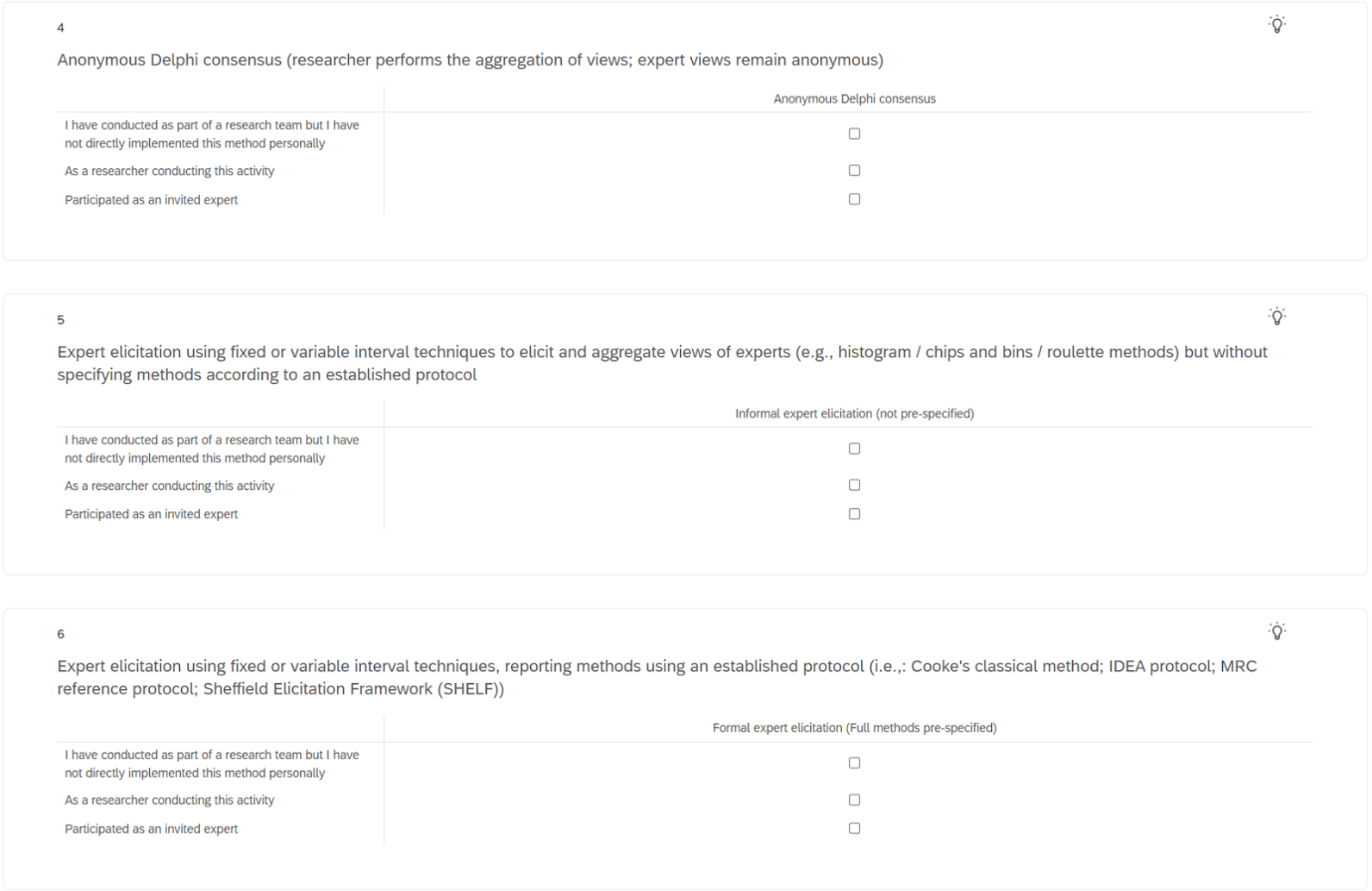


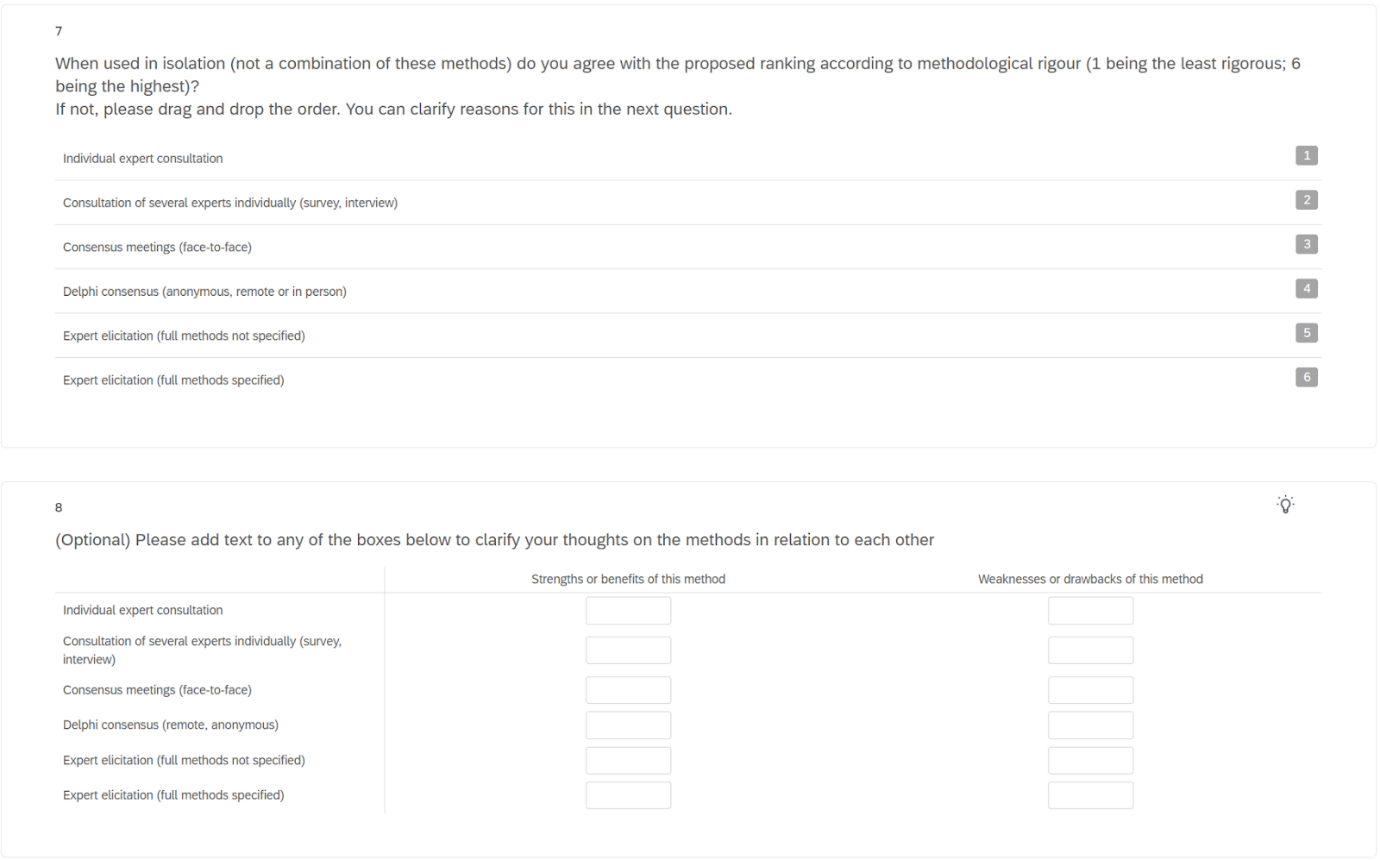


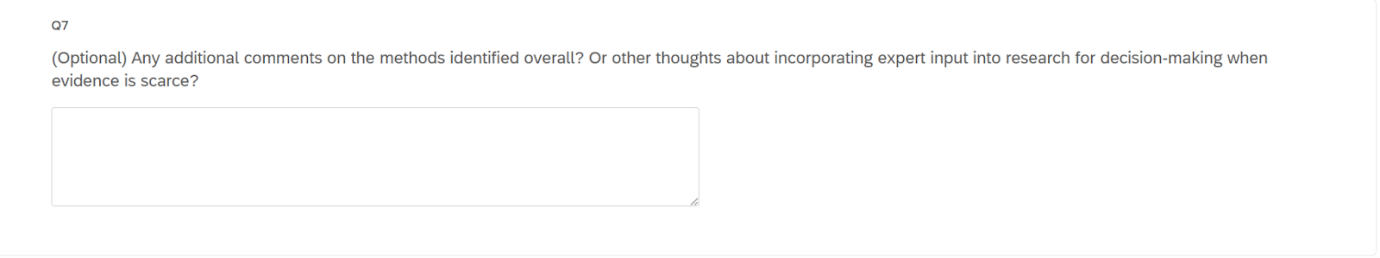


# Survey Results Summary

| **Method** | **Proposed Ranking (1 is least rigorous)** | **Mode (Survey)** |
| --- | --- | --- |
| Individual expert consultation | 1 | 1 |
| Consultation of several experts individually | 2 | 2 |
| Consensus meetings (face-to-face) | 3 | 5 |
| Delphi consensus (remote, anonymous) | 4 | 4 & 6 |
| Expert elicitation (full methods not specified) | 5 | 5 |
| Expert elicitation (full methods specified) | 6 | 6 |

When looking at the mode of the rankings alone, five of the six proposed categories received the same modal rank as suggested by the framework. However, agreement among respondents overall was very low (Kendall’s W = .04) and not statistically significant, χ²(5) = 2.27, p = 0.81. Given this lack of consistent agreement and the low number of respondents (n = 11), we favour a qualitative approach to incorporating the survey results into our recommendations, focusing instead on the strengths and drawbacks of each method highlighted by respondents. The table below summarises these.

Summary of Survey Feedback on Relative Strengths/Drawbacks to Methods

| Method | Strengths | Drawbacks |
| --- | --- | --- |
| Individual expert consultation | *“Easy to organise”*  *“Can delve deeper into a topic beyond a surface level response.”* | *“Only reflects the view of one expert” “Does not produce a consensus” “difficult to generalise from”*  *“time consuming”*  *“lack of transparency”* |
| Consultation of several experts individually | *“Can produce a lot of detail and reveal new insights”*  *“More consolidated qualitative data”*  *“Input from different experts has potential to capture most aspects that are relevant.”* | *“May not include experts from different backgrounds”*  *“Does not necessarily achieve consensus”*  *“No interaction between experts that would help when receiving conflicting input.”* |
| Consensus meetings (face-to-face) | *“Ability to hear all opinions”*  *“Allows for in-depth discussion”*  *“Helps to reduce the number of items from a delphi study to something manageable and to achieve clearer consensus” “Allows participants to learn and enhance/develop existing viewpoints.”* | *“May be dominated by those with strong opinions and voices”*  *“Expensive and requires travel”*  *“Lots of effort to organise”*  *“Usually a small group,*  *well-known, higher-status experts will likely have more to say/what they say is given more weight”* |
| Delphi consensus (remote, anonymous) | *“Able to sort through multiple ideas to find areas where consensus is likely”*  *“Large variety of opinions”*  *“All opinions weighted equally”* | *“Effort to find appropriate experts willing to take the time to respond” “Effort to ensure representativeness of experts”*  *“Not possible to discuss why something was said”*  *“Exact method used is often unclear.”* |
| Expert elicitation (full methods not specified) | *“Systematic approach (hopefully)”* | *“Results can be ‘hacked’ by altering the methods according to the input made by the experts if the methods were not pre-specified”*  *“That seems very ‘risky’ to me, because it pretends to be very systematic as methods are named”* |
| Expert elicitation (full methods specified) | *“Rigorous, systematic and transparent.”* | *“Might be more work than simply performing a descriptive analysis of interviews.”* |

The above points can be categorised as either relating to the methodology itself, or the logistics of implementing the method.

**Backgrounds**

Only 3 out of the 11 respondents reported having any involvement with the expert elicitation methodology. This lack of familiarity may have made respondents more predisposed to giving expert elicitation lower rankings than they otherwise would.
